# BPPV: Comparison of the SémontPLUS With the Sémont Maneuver: A Prospective Randomized Trial

**DOI:** 10.3389/fneur.2021.652573

**Published:** 2021-04-14

**Authors:** Michael Strupp, Nicolina Goldschagg, Anne-Sophie Vinck, Otmar Bayer, Sebastian Vandenbroeck, Lorenzo Salerni, Anita Hennig, Dominik Obrist, Marco Mandalà

**Affiliations:** ^1^Department of Neurology, German Center for Vertigo and Balance Disorders, Ludwig Maximilians University, Munich, Germany; ^2^Department of Otolaryngology, Algemeen Ziekenhuis Brugge, Brugge, Belgium; ^3^ReliaTec GmbH, Garching, Germany; ^4^Department of Otolaryngology, University of Siena, Siena, Italy; ^5^ARTORG Center for Biomedical Engineering Research, University of Bern, Bern, Switzerland

**Keywords:** BPPV [2,182], Sémont maneuver [143], Epley maneuver [477], vertigo [18,284], dizziness [35,838]

## Abstract

**Objective:** To compare the efficacy of the Sémont maneuver (SM) with the new “SémontPLUS maneuver” (SM+) in patients with posterior canal BPPV canalolithiasis (pcBPPVcan).

**Methods and Patients:** In a prospective trinational (Germany, Italy, and Belgium) randomized trial, patients with pcBPPVcan were randomly assigned to SM or SM+; SM+ means overextension of the head by 60+° below earth horizontal line during the movement of the patient toward the affected side. The first maneuver was done by the physician, and the subsequent maneuvers by the patients 9 times/day on their own. Each morning the patient documented whether vertigo could be induced. The primary endpoints were: “How long (in days) does it take until no attacks can be induced?” and “What is the efficacy of a single SM/SM+?”

**Results:** In the 194 patients analyzed (96 SM, 98 SM+), it took 2 days (median, range 1–21 days, mean 3.6 days) for recovery with SM and 1 day (median, range 1-8 days, mean 1.8 days) with SM+ (*p* = 0.001, Mann-Whitney *U*-test). There was no difference in the second primary endpoint (chi^2^-test, *p* = 0.39).

**Interpretation:** This prospective trial shows that SM+ is more effective than SM when repeated therapeutic maneuvers are performed but not when a single maneuver is performed. It also supports the hypothesis of the biophysical model: overextension of the head during step 2 brings the clot of otoconia beyond the vertex of the canal, which increases the effectivity.

**Classification of Evidence:** This study provides Class I evidence that SM+ is superior to SM for multiple treatment maneuvers of pcBPPVcan.

## Introduction

Benign paroxysmal positional vertigo (BPPV) is a very frequent cause of vertigo, with a reported prevalence of 10-140 per 100,000 and a lifetime prevalence of 2.4% (1, 2). In about 85-95% of patients, the posterior canal is affected [pc-BPPV, for reference, see (3)] with a canalolithiasis (can) as the underlying pathomechanism (4, 5). The treatment of choice is liberatory or repositioning maneuvers to remove the otoconia from the affected canal (6). The Sémont maneuver (SM) was published in 1988 (7), and the Epley maneuver in 1992 (8); both are effective (9–11).

**Figure 1:**
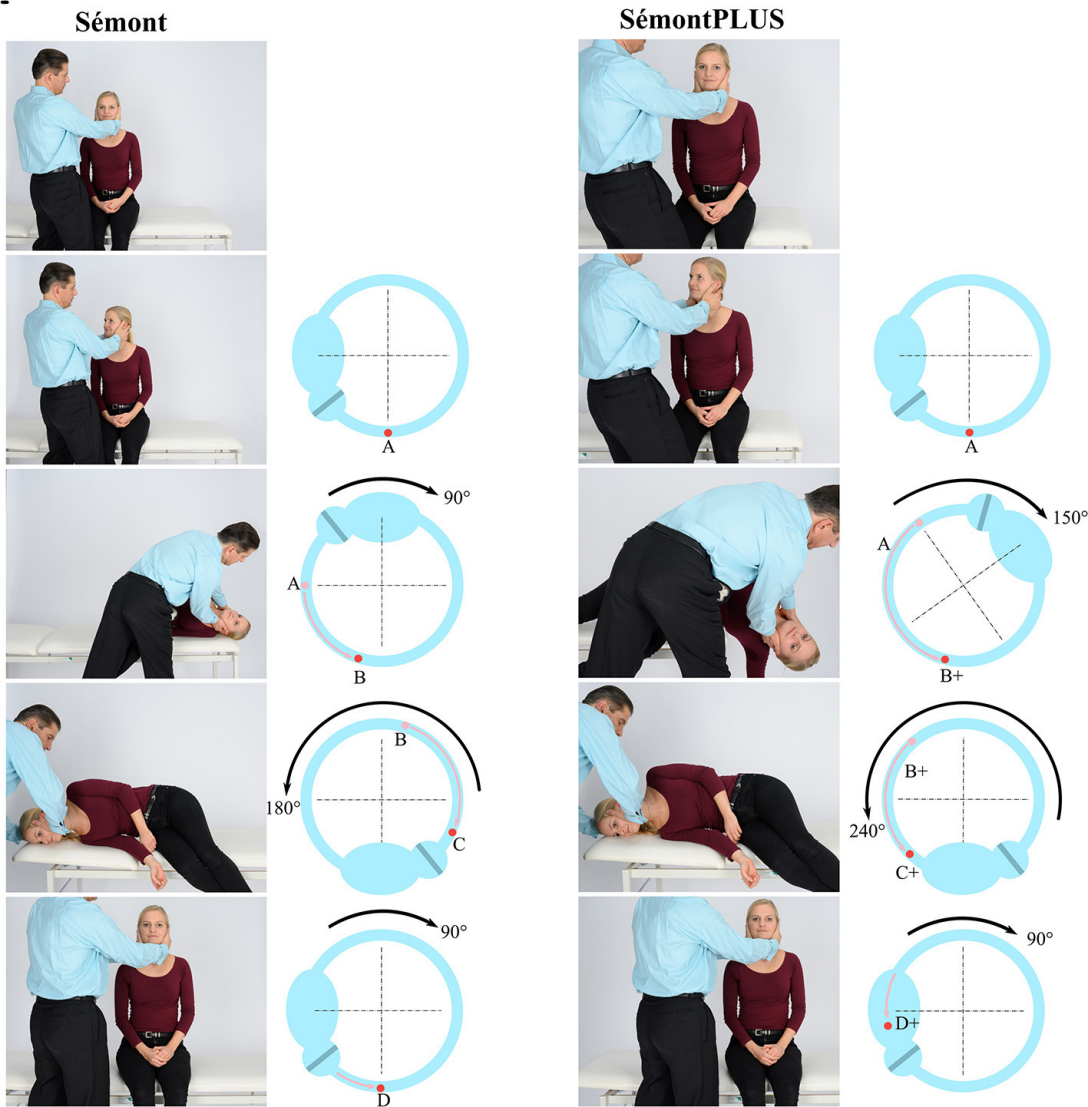
Sémont maneuver (SM, two left columns) and Sémont PLUS maneuver (SM+, two right columns with overextension of the head by 60+° toward the affected side) for the treatment of pcBPPVcan. First and third column: maneuver performed by a physician (M.S.). Second column: movement of the clot of otoconia within the left posterior canal, based on a biomechanical model of BPPV (13). **(A)** upright position; **(B)** Position of the clot after a 90° movement of the patient to the left: clot does not reach the lowest point; **(C,D)** The clot can therefore fall back into the direction of the ampulla, leading to an unsuccessful maneuver. Third column: **(A+)** upright position. **(B+)** movement of the body by 150° toward the affected side moves the otoconia farther in the direction in which they should move. **(C+)** Since the clot is beyond the vertex, the movement of body by 240° moves the clot in the direction **(D+)** of the vestibulum.

Based on our biophysical model of BPPV (12), we hypothesized that the new “SémontPLUS maneuver” (SM+) is more effective than SM because this model shows that the more the affected canal is tilted toward the affected side during the movement of the head toward the affected side, the further the otoconia move toward the exit of the posterior canal (13). This also predicts that more otoconia should then move beyond the vertex of the canal when the patient is subsequently moved toward the unaffected side (Figure 1, Supplementary Video 1). It should thus increase the effectivity of the maneuver.

In this prospective randomized trinational study, the first primary endpoint was “How long (in days) does it take until no attacks of spinning vertigo can be induced?” In this way, the effects of repeated maneuvers (three in the morning, three at noon, and three in the evening) were evaluated, with the first and second maneuvers done by the physician and the subsequent maneuvers performed by the patient—after careful instruction—as self-treatment maneuvers. This treatment regime was chosen because it reflects real world clinical practice. Further, this approach of evaluating not only the effects of a single maneuver is supported by a recent study evaluating the optimal reassessment time for treatment response in pc-BPPV (14). Finally, since symptoms typically occur and reoccur in BPPV in the morning—because otoconia may form a clot overnight, which has a higher impact on endolymphatic flow than single crystals (13, 15)—the first maneuver in the morning of each day was chosen as the first primary endpoint.

Many studies show that a single maneuver is not able to cure the majority of patients with BPPV with a wide range of reported success rates of a single SM in the literature, e.g., 37.5% (16) or 79.3% (17). Therefore, a second question was examined in this study: “Is a single SM+ more effective than a single SM?”

## Methods and Materials

### Study Population and Procedures

Patients were screened and recruited in three academic centers in three countries (Germany: Department of Neurology and German Center for Vertigo and Balance Disorders, Ludwig Maximilians University Hospital, Munich; Belgium: Department of ENT, AZ Sint-Jan Brugge, Brugge; Italy: Department of ENT, University of Siena, Siena) from June 2018 to April 2020.

Inclusion criteria were the following: eligible patients were aged > 18 years and had confirmed pcBPPVcan according to the diagnostic criteria of the International Classification Committee of Vestibular Disorders (ICVD) (1). This means that a patient's history included attacks of spinning vertigo triggered by changes in head or body positions. The duration of attacks was <1 min, accompanied by nausea, vomiting, and/or oscillopsia. The clinical findings were that when positioned to the affected ear, a patient experienced vertical-torsional nystagmus beating toward the forehead with a crescendo-decrescendo time-course lasting less than a minute.

The exclusion criteria were the following: the patient not being able to give consent; subject not wanting any treatment for BPPV; the unwillingness or inability of the patient to perform self-treatment at home.

### Study Procedures and Study Treatment

(1) Patients presented in one of the three clinics with vertigo or dizziness in the course of routine care. (2) A standard patient history was taken. Patients underwent a routine physical neurological, neuro-otological, and neuro-ophthalmological examination, including diagnostic maneuvers for BPPV. Standardized non-invasive laboratory testing with the video-head impulse test and caloric testing was performed. (3) The diagnosis of pcBPPVcan was made using the current diagnostic criteria (1). (4) The patient was informed about the study. (5) The patient gave his/her written consent. (6) Randomization (1:1) to each of the treatment groups, one-by-one in consecutive order. This was documented on a randomization list kept at each participating site, containing the number, SM or SM+, name, and date of birth of the patient. (7) SM or the SM+ (Figure 1 and Supplementary Video 1 of SM+) was carried out once. The angle of the head was measured using an AppStore App (“Kompass”) installed on iPhones, which can also be used as an inclinometer, so that standardized examination conditions were guaranteed. The Sémont maneuver means horizontal, i.e., 0°; SM+ means 60° beyond earth horizontal; each of the three positions (with the head turned toward the unaffected side) was maintained for 60 s: (1) movement of the patient's body toward the affected side; (2) movement of the body toward the unaffected side; (3) sitting upright. Fifteen to sixty minutes after the first therapeutic maneuver, a second diagnostic maneuver was performed to check the effect of the first maneuver, i.e., whether positional vertigo and/or positional nystagmus can be induced. Depending on randomization to SM or SM+, the patient independently carried out SM or SM+ three times in the morning, three times at noon, and three times in the evening as instructed. The patient noted how many days after the start of the SM or SM+ maneuver it took for him/her to no longer experience positional vertigo. The time point was the first maneuver in the morning of the day on which the patient was not able to induce positional spinning vertigo. This was documented by the patient using a standardized evaluation sheet. On the day of inclusion, patients received a written form with a standardized questionnaire and an envelope that they had to send back to the center.

### Endpoints

Two primary endpoints were chosen to evaluate two questions: (1) the long-term effect of SM vs. SM+, i.e., the “real world recovery” for the patient and (2) the short-term effect of a single SM vs. SM+ because of the wide range of reported efficacy of single treatment maneuvers in the literature (see **Introduction**).

The first primary endpoint is How long (in days) it takes until no attacks of spinning vertigo can be induced “in the morning” by the maneuvers. Day 0 was the day of the examination and the first liberatory maneuver in the hospital, day 1 the next morning. For a maneuver to be rated as successful, the patient should not be able to induce positional vertigo in three consecutive maneuvers.

The second primary endpoint is the success rate of a single liberatory maneuver, i.e., either SM or SM+, on the occurrence of vertigo and/or positional nystagmus, tested after the first SM or SM+ (“yes” or “no”). If neither spinning vertigo nor nystagmus was induced in the diagnostic maneuver, the liberatory maneuver was rated as primarily successful. If vertigo and/or nystagmus were detected, it was rated as primarily unsuccessful.

### Randomization

Patients who met the eligibility criteria for enrollment were randomized in a 1:1 ratio to receive either SM or SM+.

### Statistical Analysis

Statistical analysis and graphic design were performed using R version 3.5.2 (the R Foundation for Statistical Computing, www.r-project.org). Since days to recovery were not normally distributed, non-parametric testing using the Mann-Whitney *U*-test was performed. To compare treatment success after the first maneuver, a chi-square test was applied. Differences were considered significant if *p* < 0.05.

### Sample Size Calculation

To detect an improvement of the success rate (first primary endpoint) from 0.50 to 0.70 with a power of 0.80 on a significance level (one-sided testing) of *p* = 0.05, at least 93 analyzable patients are required in each group, resulting in a total number of at least 186 analyzable patients.

### Standard Protocol Approvals, Registrations, and Patient Consents

The study was performed in accordance with the Helsinki II Declaration. The study protocol, including the patient information and consent form, was approved by the local ethics committee of each participating institution (Leading ethics committee: Ethics committee of the Medical Faculty of the Ludwig Maximilians University, Munich, Germany; reference number: 17-477, and subsequently by the Ethics committee of AZ ST JAN, Brugge Oostende, Belgium, AV Ethics committee OG 065, BUN: 8049201835209 Int. Nr.2247, which required modifications of the protocol; date of final approval: May 17th, 2018). All participants gave written informed consent.

## Data Availability Policy

Upon request, further data including the study protocol will be shared with other investigators for the purpose of replicating procedures and results. Unidentified participant data may not be shared for legal or ethical reasons. Data cannot be shared publicly because participants did not explicitly consent to the sharing of their data as per the European Union's General Data Protection Regulation and the corresponding German privacy laws. Data are available through the Research Ethics Board of Ludwig Maximilians University, Munich, Germany, for researchers who meet the criteria for access to confidential data.

## Results

### Study Population

**Figure 2:**
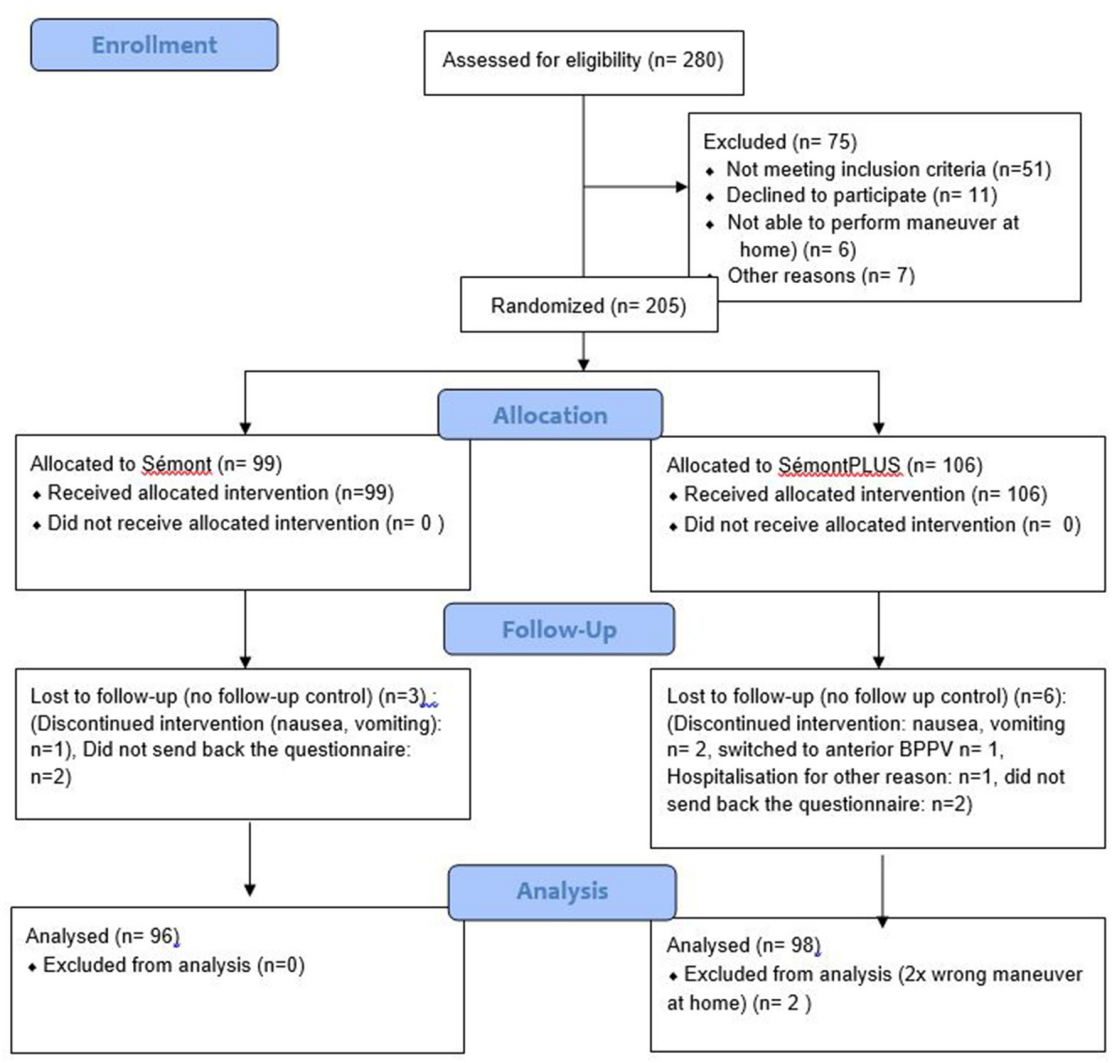
Trial profile: Consort flow diagram.

In the three centers, a total of 280 patients were assessed for eligibility (Figure 2; CONSORT flow diagram); 75 were excluded (51 did not meet the inclusion criteria, 11 declined to participate, 6 were not able to perform the maneuvers at home, and 7 were excluded for other reasons), so that 205 patients with pcBPPVcan were randomized. Ninety-nine were allocated to the SM and 106 to the SM+ group. Three patients were lost to follow-up in the SM group, and six patients were lost in the SM+ group. A total of 194 patients were finally analyzed: 96 patients in the SM group and 98 in the SM+ group. The mean age of the patients in the SM group (60 females) was 64 years (range 19–87 years); in the SM+ group (58 females) it was 63 years (range 19–90 years). In the SM group, 62 of 96 and in the SM+ group 62 of 98 had right pc-BPPV (Table 1).

**Table 1:** Patient Characteristics.

	**Allocated to the Sémont maneuver**	**Allocated to the Sémont Plus maneuver**
Mean age (± SD), range	64 ± 13 years, 19–87 years	63 ± 13 years, 19–90 years
Sex: male/female	36/60	40/58
Affected side R/L	62/34	62/36
Etiology: idiopathic/other/missing data	79/15/2	85/12/1
First episode of BPPV/Recurrent BPPV/Missing data	54/40/2	60/37/1
Mean duration of symptoms before		
inclusion in the study:		
Median (in days)	7	5
Range (in days)	1–7200	1–5470
Missing data	11	6

### Outcomes

**Figure 3:**
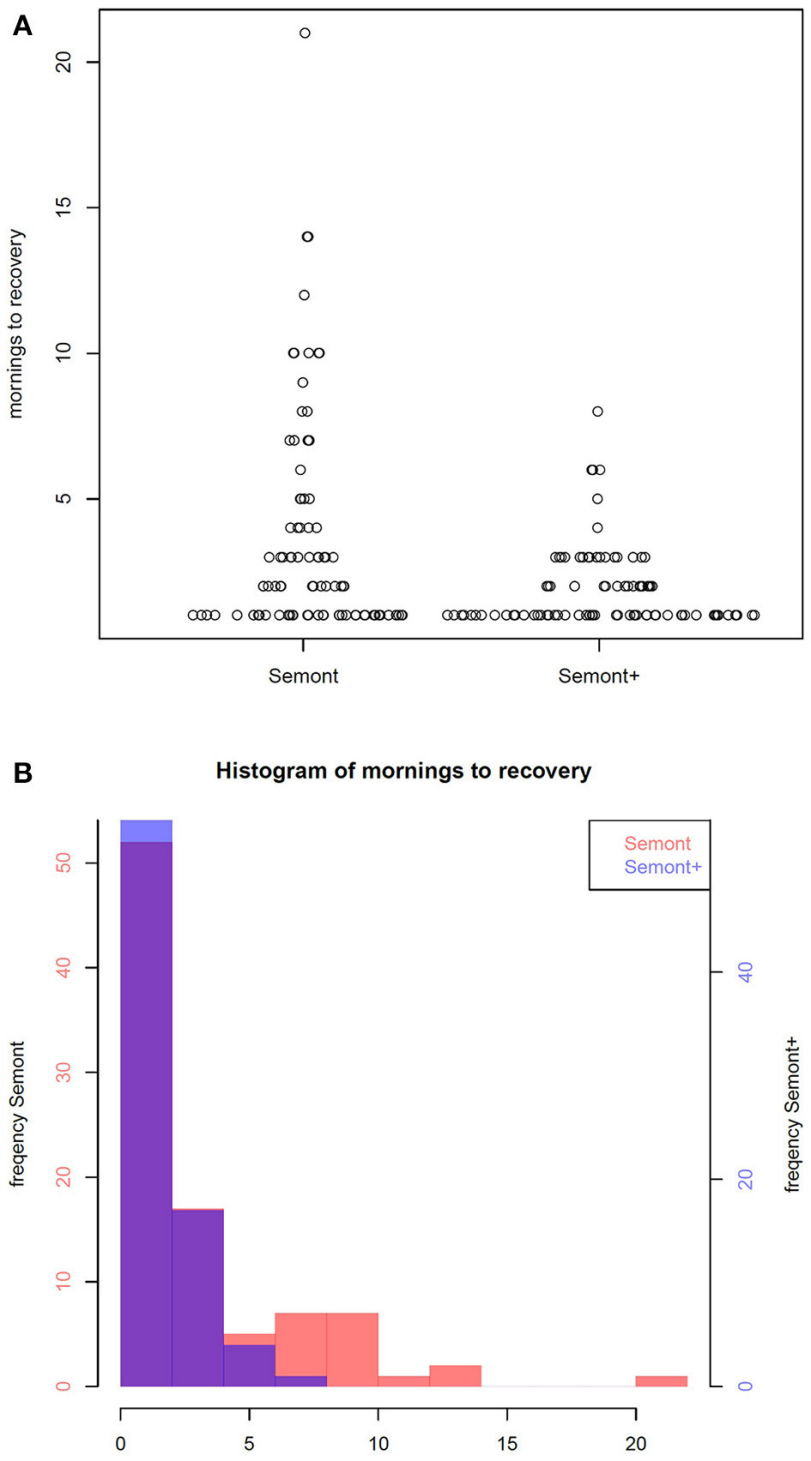
Comparison of the Sémont with the SémontPLUS maneuver. **(A)** Scatter plot of the time to recover for the Sémont (left) and the SémontPLUS maneuver (right) in days. **(B)** Histogram of time to recover for the Sémont (red) and the SémontPLUS maneuver, purple, which is due to the overlay of red (SM) and blue (SM+), in days.

The first primary endpoint is how long (in days) it takes until no attacks of spinning vertigo can be induced “in the morning” by the maneuvers. In the SM group, it took 2 days for recovery (median, range 1–21 days, mean 3.6 days). In the SM+ group, it took 1 day (median, range 1–8 days, mean 1.8 days) for recovery (*p* = 0.001, Mann-Whitney *U*-test) (Figure 3).

The second primary endpoint is the success rate of a single liberatory maneuver, i.e., either SM or SM+, on the occurrence of positional spinning vertigo and/or positional nystagmus, tested after the first SM or SM+ maneuver (“yes” or “no”).

In the SM group, 46 out of 95 patients −48% (95% CI: 38–59%)—had neither positional vertigo nor positional nystagmus after the first maneuver. In the SM+ group, 54 out of 97 patients-−56% (95% CI: 45–66%)—were free of symptoms after the first maneuver. There was no statistical difference for the second primary endpoint (effect of a single maneuver) (chi-square test, *p* = 0.39).

## Discussion

The major findings of this randomized prospective trinational study are as follows:

First, in the performance of multiple liberatory maneuvers for the treatment of pcBPPVcan SM+ significantly reduces the time until patients are free of attacks of vertigo by about 50%. These findings are in agreement with and support prior results from biophysical studies on BPPV (13): the farther the head is turned toward the unaffected side during the movement of the body toward the unaffected side, the higher the efficacy of the liberatory maneuver. This was already suggested earlier with an overextension by 15° (6) and is now proven by this trial.

Second, the immediate success rate of a *single* maneuver was low, and there was no difference between the success rate of a single SM (48%) and a single SM+ (56%). This finding is in line with previous studies showing that a single maneuver is not sufficient for a successful treatment of pc-BPPV (16, 17). Therefore, the study was designed to evaluate the efficacy of multiple maneuvers, thereby also reflecting real world procedures for the treatment of BPPV in clinical practice: the combination of the first maneuver by the therapist in the office and subsequent self-maneuvers by the patient at home after receiving detailed instructions on how to perform the maneuvers, which was also developed, used, and recommended in other studies (18–21). At least for the Epley maneuver, the efficacy of the self-maneuver was shown to be higher than that of the Epley maneuver alone (22).

In a previous study, three SM per day with self-treatment were performed and after 1 week 58% of patients were cured (18). In our study with nine SM maneuvers per day, 57% were cured after only 2 days; after 1 week of nine SM per day 86% were free of symptoms. This comparison shows that the number of maneuvers per day also seems to be relevant. Therefore, we would suggest nine instead of three per day.

Finally, since SM+ is more difficult to perform than SM, it is possible that the study might have underestimated the efficacy of SM+. On the other hand, SM+ may not be suitable for all patients because it requires more skills than the regular SM. Therefore, the choice of maneuver to be used should be made on an individual basis.

In an on-going study with a similar design, the effects of SM+ are compared with the Epley maneuver (Project number 20-072). Furthermore, based on our findings, the efficacy of the diagnostic Sémont maneuver will be compared with the *diagnostic SémontPLUS maneuver* (dSM+) with an overextension by 60°, which should theoretically be more effective as well.

## Limitations

This study has several limitations: first, one of the endpoints was based on a self-reported outcome by the patient and not by re-examination of the patient by a physician. However, patients received detailed instructions and used a standardized questionnaire. Furthermore, since symptoms typically first occur and re-occur in the early morning and then improve during daytime, a reevaluation in the hospital may also give false normal findings because patients might have already transiently recovered before reaching the hospital. Finally, treatment of the patient with benign PPV on a ward until recovery or recurrent daily visits to document the treatment effects is not practical and again does not reflect real world procedures. Second, in this study only the effects of a single therapeutic maneuver in combination with recurrent self-maneuvers by the patients were evaluated. Therefore, we cannot make a statement about the efficacy of repeated maneuvers performed by physicians or physiotherapists. Third, we did not specifically evaluate the side effects of both maneuvers or the impact of the maneuvers on quality of life or functioning.

## Conclusion

This prospective randomized trial provides Class I evidence that SM+ is more effective than SM for the treatment of pcBPPVcan when repeated therapeutic self-maneuvers are performed but not when a single maneuver is performed. This is in line with the findings of the biophysical model: overextension of the head during step 2 of SM+ brings the clot of otoconia beyond the vertex of the canal, which increases the efficacy. Therefore, for clinical practice SM+ can be recommended for most patients.

## Data Availability Statement

The raw data supporting the conclusions of this article will be made available by the authors, without undue reservation.

## Ethics Statement

The studies involving human participants were reviewed and approved by Leading ethics committee: Ethics committee of the Medical Faculty of the Ludwig Maximilians University, Munich, Germany; reference number: 17-477 and subsequently by the Ethics committee of AZ ST JAN, Brugge Oostende, Belgium, AV Ethics committee OG 065, BUN: 8049201835209 Int. Nr.2247, which required modifications of the protocol; date of final approval: May 17th, 2018. The patients/participants provided their written informed consent to participate in this study. Written informed consent was obtained from the individual(s) for the publication of any potentially identifiable images or data included in this article.

## Author Contributions

MS: idea for the study, conception, writing of the protocol, recruitment and examination of the patients, interpretation of the data, and drafting the manuscript. NG: interpretation of the data and drafting the manuscript. A-SV, SV, LS, and MM: recruitment and examination of the patients, interpretation of the data, and drafting the manuscript. OB: writing of the protocol, statistical design, sample size calculation, statistical analysis, interpretation of the data, and drafting the manuscript. AH: recruitment of patients, statistical analysis, interpretation of data, and drafting the manuscript. DO: idea for the study, conception, interpretation of data, and drafting the manuscript. All authors contributed to the article and approved the submitted version.

## Conflict of Interest

MS has received speaker's honoraria from Abbott, Actelion, Auris Medical, Biogen, Eisai, Grünenthal, GSK, Henning Pharma, Interacoustics, MSD, Otometrics, Pierre-Fabre, TEVA, UCB, and Viatris. MS is a shareholder of IntraBio. MS is the distributor of M-glasses and the Positional vertigo App. MS acts as a consultant for Abbott, Actelion, AurisMedical, Heel, IntraBio, and Sensorion. NG received honoraria from IntraBio outside of this study. OB was employed by Relia Tec GmbH. The remaining authors declare that the research was conducted in the absence of any commercial or financial relationships that could be construed as a potential conflict of interest.

## Supplementary Material

Supplementary Video 1 SémontPLUS maneuver for right posterior canal BPPV. The overextension (with the right arm extended) is essential. Each position should be maintained for 60 s so that the otoconia can reach the lowest point relative to gravity.
